# Data for new protocol to detect the monoclinic phase of La_2_Mo_2_O_9_ and related oxide ion conductors

**DOI:** 10.1016/j.dib.2018.04.078

**Published:** 2018-04-30

**Authors:** Tanmoy Paul, Yoed Tsur

**Affiliations:** Department of Chemical Engineering and the Grand Technion Energy Program, Technion Israel Institute of Technology, Haifa 3200003, Israel

## Abstract

In this data article we have presented the Rietveld refinement results of the X-ray diffraction data of both bulk and nanocrystalline La_2_Mo_2_O_9_ and bulk La_2_MoWO_9_ oxide ion conductors at different temperatures. Bulk and nanocrystalline La_2_Mo_2_O_9_ samples were prepared by conventional solid state reaction and glycine auto ignition method, respectively. Transmission electron microscopy is also employed to determine the grain size of the sample prepared via glycine auto ignition method.

**Specifications table**

**Table:** 

Subject area	*Physics*
More specific subject area	*Structure and thermal properties of materials for energy*
Type of data	*Images (X-ray, trasmission electron microscopy), thermogravimetry*
How data was acquired	*X-ray diffraction (Rigaku, Smartlab); TG/DTA (TA instruments, Q600); TEM (Technai, T20)*
Data format	*Raw and analyzed*
Experimental factors	*For X-ray diffraction, both Bragg-Brentano and parallel beam methods are used*
Experimental features	*XRD data are taken at different temperatures with fixed scan rate*
Data source location	*Technion-Israel Institute of Technology, Haifa, Israel*
Data accessibility	*Data is with this article*

**Value of the data**

• The data is given to supplement our short manuscript [1].
• Dense nano-ceramic samples are prepared by glycine auto ignition method.
• Rietveld refinements of the XRD patterns of bulk and nanostructured La_2_Mo_2_O_9_ and La_2_MoWO_9_ at several temperatures are presented.
• Thermogravimetry shows the α/β phase transition.

## 1 Data

Temperature dependent X-ray diffraction data were obtained using a Rikagu SmartLab 9 kW high-resolution diffraction system. The pellets of La_2_Mo_2_O_9_ and La_2_WMoO_9_ oxide ion conductors were taken for data acquisition in Bragg Brentano method with *K*_β_ filter, while for powder sample, the parallel beam method was used.

## 2 Experimental design, materials and methods

**Fig. 1:**
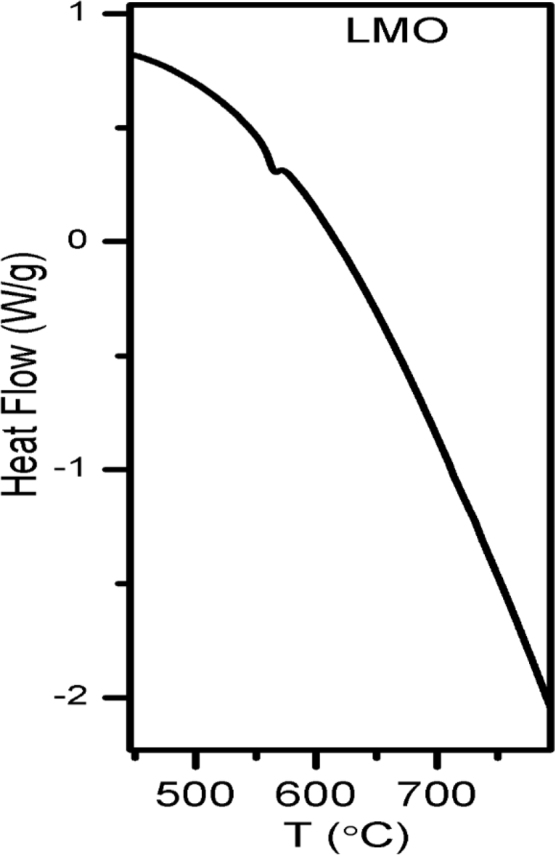
TG/DTA thermogram for bulk La_2_Mo_2_O_9_.

**Fig. 2:**
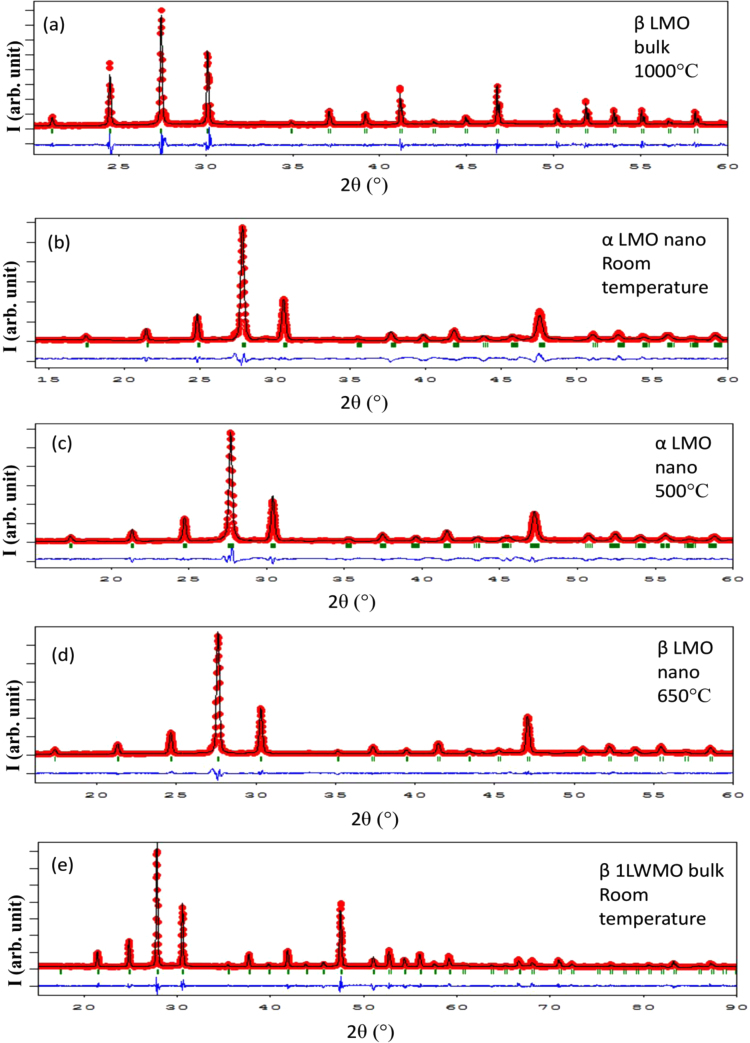
Rietveld refinements of the X-ray diffraction patterns for (a) bulk La_2_Mo_2_O_9_; (b) nanocrystalline La_2_Mo_2_O_9_ at room temperature; (c) nanocrystalline La_2_Mo_2_O_9_ at 500 °C; (d) nanocrystalline La_2_Mo_2_O_9_ at 650 °C and (e) bulk La_2_MoWO_9_ at room temperature. The Red symbols are observed patterns; the Black lines are calculated and the Blue lines are the difference between them. The vertical bars represent the Bragg positions. ‘I’ stands for the intensity.

**Fig. 3:**
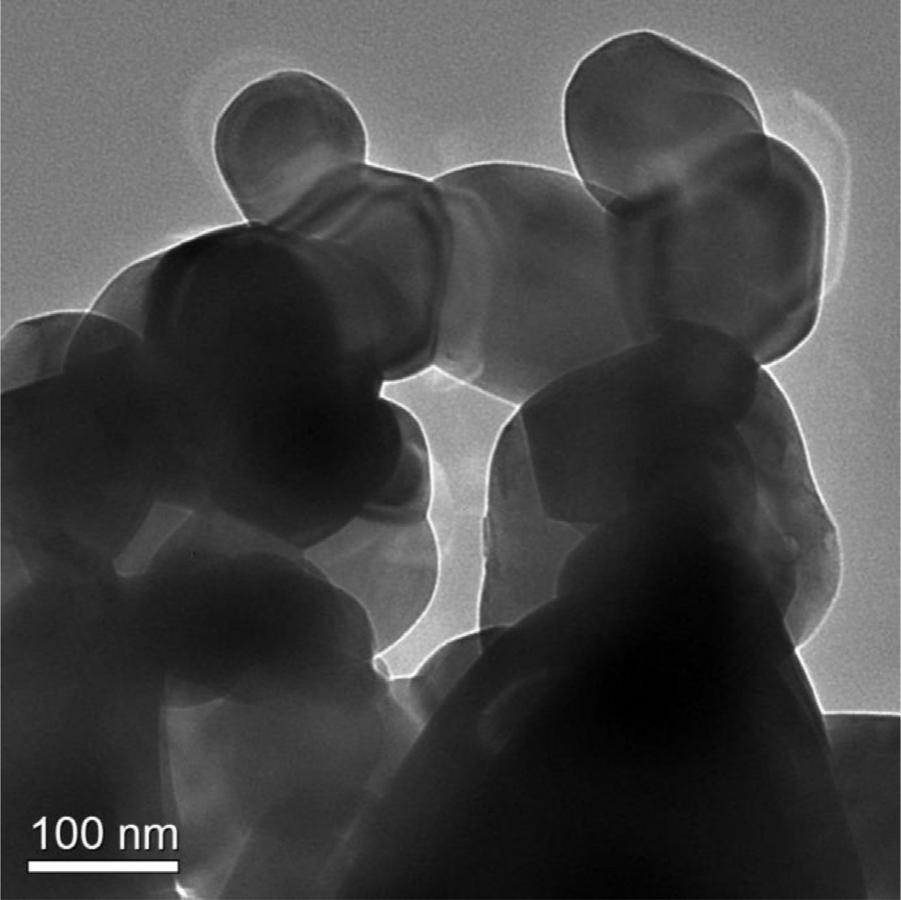
The HRTEM image of nanocrystalline La_2_Mo_2_O_9_ prepared by glycine auto ignition method.

In solid state reaction method the stoichiometric amounts of La_2_O_3_ (Sigma Aldrich, Lot No MKBS7302V) and MoO_3_ (Strem Chemicals, Lot No. 30342600) powders having purity 99.99% and 99.999% were mixed in high pure ethanol for 12 h with 12 YSZ balls in a plastic bottle. Then the mixtures were heated at 80 °C for drying. The dried mixtures were heated at 500 °C for 12 h and after cooling pressed into pellets at 375 MPa and sintered at 1000 °C for 12 h. In glycine auto ignition method, powders of LaN_3_O_9_ (Sigma Aldrich, Lot No MKBV6268V; Purity 99.99%) and (NH_4_)_6_Mo_7_O_24_·4H_2_O (Alfa Aesar, Lot No. 10199521; Purity 99%) were taken as starting materials in their appropriate ratios and were thoroughly mixed in aqueous solution of glycine (Sigma Aldrich, Lot No. 1154851; Purity 99.5%). The glycine was used as a chelating agent and the molar ratio of the glycine to that of the cations was taken as 2:1. The mixed solution was stirred on a magnetic stirrer for about 6 h; the temperature of the solution was maintained at around 80 °C. The water was evaporated slowly with heating and after several minutes, auto ignition was taking place, resulting with a black precursor. The precursor was precalcined at 200 °C for 2 h to remove the organic solvent completely. The dried mixtures was heated in an electric furnace at 800 °C for 5 h. To prepare La_2_MoWO_9_ oxide ion conductor we took WO_3_ (Sigma Aldrich, Lot No BCBR3791V, Purity 99.9%) and followed the similar process as described for that of the bulk. The calcined powder was heated at 1100 °C and finally sintered at 1100 °C for 12 h in pellet form. During all the heating treatments, the ramp rate was kept fixed at 3 °C/min. The thermal property of the bulk La_2_Mo_2_O_9_ powder was investigated in a thermobalance (TA instruments, SDT Q600). For thermal property investigation a powder sample of ~30 mg was placed in a platinum crucible in N_2_ atmosphere with 100 ml/min as purge. Fig. 1 shows the thermogram for bulk La_2_Mo_2_O_9_. The crystal symmetry of all samples was checked in an X-ray diffractometer at different temperatures as presented in Fig. 2. The interpretation of the result is described in Ref. [1]. For transmission electron microscopy study, the powder of nanocrystalline LMO sample was dispersed in ethanol for several hours and poured into a 400 mesh holey carbon coated copper grid. Fig. 3 shows different grains of size ~50 nm.
