# Supplementary material for: Data for new protocol to detect the monoclinic phase of La2Mo2O9 and related oxide ion conductors
Source: Data Brief. 2018 Apr 30;18:1637–41. doi: 10.1016/j.dib.2018.04.078 (PMC5998216; doi:10.1016/j.dib.2018.04.078)
Supplement: Supplementary file 1 — Supplementary material [file mmc1.rtf]

Conflict of Interest
There is no conflict of interest.
